# Supplementary material for: Unstructured clinical notes within the 24 hours since admission predict short, mid & long-term mortality in adult ICU patients
Source: PLoS One. 2022 Jan 6;17(1):e0262182. doi: 10.1371/journal.pone.0262182 (PMC8735614; doi:10.1371/journal.pone.0262182)
Supplement: Attachment — Submitted filename: Response to Reviewers.pdf [file pone.0262182.s001.pdf]

Dear Dr. Wajid Mumtaz,

Thank you for giving us the opportunity to resubmit our revised manuscript “Unstructured clinical notes within the 24 hours since admission predict short, mid & long-term mortality in adult ICU patients”, Manuscript. No: PONE-D-21-29049 to PLOS ONE. In this version of the manuscript, we have carefully addressed the comments and concerns raised by you and the reviewer and made changes throughout the manuscript accordingly. We appreciate the time and effort that you and the reviewer have dedicated to providing feedback on our manuscript. Below, we detail how we have responded to the comments. We also want to add that in the ‘Revised Manuscript with Track Changes’, we have highlighted the edited/amended texts and struck through the deleted texts.

Sincerely,
Maria Mahbub

Reviewer Points:

- Why not use both unstructured and structured clinical data? As the authors note, their is some overlap between these entities, and also some unique information that is contained only in unstructured data and some that is contained only in unstructured data. Unstructured data has a theoretical advantage of greater objectivity. As you may know, a group from Boston and industry have shared the perspective that machine learning applications often learn clinicians' thought processes even when using structured data, producing outputs that tell the clinician that which they already knew (PMID: 33785839). Unstructured data from clinical notes seems to reflect clinicians' thought process to an even greater degree. Please discuss these perspectives and justify the use of clinical notes alone in prediction modeling.

Answer: We agree with the reviewer. As the unstructured data occupy 80% of the EHR, they can capture more detailed information on patients, resulting in better performance of machine learning models. As such, models trained on these data may better capture the thought process of the clinicians and the conditions of the patients compared to models trained on structured data. However, to conclude the advantages of using unstructured clinical notes over structured data for predictive modeling in our problem setting, we need to further experiment with only structured data as well as both structured and unstructured data. But, since the focus of this study is to unveil the potential of raw unstructured clinical notes for predicting mortality in adult ICU patients, we use solely unstructured clinical notes in prediction modeling for this work and leave the comparative analysis for future work.

We added a brief discussion on these perspectives of using only clinical notes in prediction modeling in the *Introduction* section of the paper.

Editorial comments:

Journal requirements:

1. Please ensure that your manuscript meets PLOS ONE's style requirements, including those for file naming.

Answer: We revised the manuscript to meet the style requirements of the journal, including section headings, figure captions and references, and table captions and references.

2. Please state what role the funders took in the study. Please include this amended Role of Funder statement in your cover letter; we will change the online submission form on your behalf.

Answer: We have added the role of funders in the study. Here is the amended Role of Funder statement: "The funders had no role in study design, data collection and analysis, decision to publish, or preparation of the manuscript."

3. Please remove any funding-related text from the manuscript and let us know how you would like to update your Funding Statement. Please include your amended statements within your cover letter; we will change the online submission form on your behalf.

Answer: We have removed all funding-related texts from the manuscript. We have removed the acknowledgment section from the manuscript and would like to update the Funding statement in the online submission form. Here is the amended statement: **"**This research used resources of the Knowledge Discovery Infrastructure at the Oak Ridge National Laboratory, which is supported by the Office of Science of the U.S. Department of Energy under Contract No. DE-AC05-00OR22725 and the Department of Veterans Affairs Office of Information Technology Inter-Agency Agreement with the Department of Energy under IAA No. VA118-16-M-1062. This manuscript has been in part co-authored by UT-Battelle, LLC under Contract No. DE-AC05-00OR22725 with the U.S. Department of Energy. The United States Government retains and the publisher, by accepting the article for publication, acknowledges that the United States Government retains a non-exclusive, paid-up, irrevocable, world-wide license to publish or reproduce the published form of this manuscript, or allow others to do so, for United States Government purposes. The Department of Energy will provide public access to these results of federally sponsored research in accordance with the DOE Public Access Plan (http://energy.gov/downloads/doe-public-access-plan). The funders had no role in study design, data collection and analysis, decision to publish, or preparation of the manuscript. The specific roles of all authors are articulated in the 'author contributions' section.**"**

4. PLOS requires an ORCID iD for the corresponding author in Editorial Manager on papers submitted after December 6th, 2016. Please ensure that you have an ORCID iD and that it is validated in Editorial Manager.

Answer: We have added and validated the ORCID iD for the corresponding author in the Editorial Manager.

5. Your ethics statement should only appear in the Methods section of your manuscript. If your ethics statement is written in any section besides the Methods, please delete it from any other section.

Answer: We have edited the ethics statement in the Methods section of our manuscript and removed it from the other section(s).

6. Please review your reference list to ensure that it is complete and correct. If you have cited papers that have been retracted, please include the rationale for doing so in the manuscript text, or remove these references and replace them with relevant current references. Any changes to the reference list should be mentioned in the rebuttal letter that accompanies your revised manuscript. If you need to cite a retracted article, indicate the article's retracted status in the References list and also include a citation and full reference for the retraction notice.

Answer: We have reviewed the reference list to check for quality. We have updated several references to be more complete.

7. While revising your submission, please upload your figure files to the Preflight Analysis and Conversion Engine (PACE) digital diagnostic tool.

Answer: We have used PACE to make appropriate corrections to the figure files. We have uploaded the corrected figure files.
